# The clinicopathological and prognostic impact of 14-3-3 sigma expression on vulvar squamous cell carcinomas

**DOI:** 10.1186/1471-2407-8-308

**Published:** 2008-10-24

**Authors:** Zhihui Wang, Claes G Tropè, Zhenhe Suo, Gunhild Trøen, Guanrui Yang, Jahn M Nesland, Ruth Holm

**Affiliations:** 1 Division of Pathology, The Norwegian Radium Hospital, Rikshospitalet University Hospital, Oslo, Norway; 2 Department of Oncology, The First Affiliated Hospital of Zhengzhou University, Medical College of Zhengzhou University, Zhengzhou, PR China; 3 Faculty Division The Norwegian Radium Hospital, Medical Faculty, University of Oslo, Norway; 4 Division of Obstetrics and Gynaecology, The Norwegian Radium Hospital, Rikshospitalet University Hospital, Oslo, Norway; 5 Henan Institute of Medical Sciences, Medical College of Zhengzhou University, Zhengzhou, PR China

## Abstract

**Background:**

14-3-3 sigma (σ) promotes G2/M cell cycle arrest by sequestering cyclin B1-CDC2 complex in cytoplasm. Down-regulation of 14-3-3σ, which has been demonstrated in various carcinomas, may contribute to malignant transformation. However, the exact role of 14-3-3σ in the pathogenesis of vulvar carcinoma is not fully characterized, and the prognostic impact of 14-3-3σ protein expression is still unknown.

**Methods:**

We investigated the 14-3-3σ expression in a series of 302 vulvar squamous cell carcinomas using immunohistochemistry and its associations with clinicopathological factors and clinical outcome.

**Results:**

In cytoplasm, nucleus and cytoplasm/nucleus of vulvar carcinomas high 14-3-3σ protein expression was found in 72%, 59% and 75% of the carcinomas, respectively, and low levels in 28%, 41% and 25% of the cases, respectively. High level of 14-3-3σ in cytoplasm, nucleus and cytoplasm/nucleus was significantly correlated to large tumor diameter (*p *= 0.001, *p *= 0.002 and *p *= 0.001, respectively) and deep invasion (*p *= 0.01, *p *= 0.001 and *p *= 0.007, respectively). Variations of 14-3-3σ protein expression were not associated to disease-specific survival.

**Conclusion:**

Our results indicate that 14-3-3σ may be involved in the development of a subset of vulvar squamous cell carcinomas by down-regulation of 14-3-3σ protein. Neither cytoplasmic nor nuclear level of 14-3-3σ expression was associated with prognosis.

## Background

Vulvar carcinoma represents 3–5% of all female genital cancer [1] and was previously considered a disease of elderly women. However, we see an increase in the incidence of vulvar carcinomas among younger women in recent years [2]. Considerable morbidity is often followed radical surgery, which has been the standard treatment [3]. Individualized therapy has been introduced to raise the quality of life for vulvar cancer patients [4]. Targeted therapy urge us to search for factors involved in tumorigenesis as well as identification of new tumor markers for therapy.

In mammals, 14-3-3 proteins are composed of a highly conserved multigene family of small acidic proteins, including seven 14-3-3 isoforms (β, ε, γ, η, σ, τ, ζ) [5]. As a phosphoserine/phosphothreonine-binding protein, 14-3-3s bind to proteins within their RS*X*pS*X*P and R*X*(Y/F) *X*pS*X*P sequences (pS represents phosphoserine or phosphothreonine) [6] and thereby play an important role in various cellular processes such as altering enzymatic activity, subcellular localization, protein-protein interactions, dephosphorylation, and proteolysis of individual target proteins [7-9].

14-3-3σ, also named stratiffin, is mostly expressed in human epithelial cells [8]. Responsive to DNA damage, 14-3-3σ expression is induced in a p53-dependent manner [10-12]. 14-3-3σ sequesters cyclin B1-CDC2 complex in cytoplasm, and prevents its entry into nucleus resulting in G2/M cell cycle arrest [10,12]. Furthermore, 14-3-3σ can link to CDK2 and CDK4 and block the cell cycle entry [13]. Thus, 14-3-3σ acts as a negative regulator of cell cycle progression and is linked to cancer [14].

Epigenetic change in the 14-3-3σ gene by CpG hypermethylation followed by down-regulation of 14-3-3σ expression has been demonstrated in various carcinomas [15-22]. Furthermore, in breast carcinoma [22], endometrial carcinoma [23] and colorectal carcinoma [24] 14-3-3σ expression was found to be associated with the patients' clinical outcome. In a previous study, including a limited number of vulvar carcinomas, appropriately 60% of the cases revealed CpG methylation in the 14-3-3σ gene [25]. However, the expression of the 14-3-3σ protein and its connection with prognosis has still not been reported in vulvar carcinoma patients. Thus, we intended to study the 14-3-3σ expression in a series of 302 vulvar squamous cell carcinomas in order to disclose the correlation between 14-3-3σ expression and its prognostic value.

## Methods

### Patient materials

A retrospective study was performed, in which 302 patients with squamous cell carcinoma of vulvar underwent surgery at The Norwegian Radium Hospital in the period 1977–2006. Before surgery, radiotherapy was given to 9 (3%) of the patients, additionally, 3 (1%) of the patients received chemotherapy. Radical vulvectomy was performed on 201 (67%) of the patients. Postoperative irradiation was given to 63 (21%), chemotherapy to 3 (1%) and irradiation/chemotherapy to 4 (1%) of the patients. One hundred and eight (36%) patients suffered from a relapse. The median age at diagnosis was 74 years (range 35–96 years). All patients were followed until death or 31. December, 2006. One hundred and twenty patients (40%) died of vulvar cancer. The median follow-up time for all patients was 52 months (range 0.4–346 months), whereas the median follow-up time for patients alive at last observation was 131 months (range 11–346 months). All tumors were staged according to the International Federation of Gynecology and the Obstetrics (FIGO) classification [26]. The Regional Committee for Medical Research Ethics South of Norway (S-06012), The Social- and Health Directorate (04/2639 and 06/1478) and The Data Inspectorate (04/01043) approved the study.

Histological specimens were reviewed by one of the authors (J.M.N.) who had no access to clinical information. The tumors were classified according to World Health Organization recommendations [27] and 284 (94%) were keratinizing/nonkeratinizing, 14 (5%) were basaloid and 4 (1%) were veruccoid. The primary tumors of the patients have previously been investigated for the expression of p53, p16, p21, p27, p14, and the cyclins A, D1, D3 and E and human papillomavirus (HPV) [28-31]. Table 1 provides a detailed description of the tumor characteristics. As controls, samples of normal vulva were collected form 11 patients undergone surgery for benign gynecological diseases.

**Table 1:** 14-3-3σ immunostaining in relation to clinicopathological variables

Variables	Total	Cytoplasm				Nucleus				Cytoplasm and nucleus			
				
	n	Low	High	(%)	*p*^1^	Low	High	(%)	*p*^1^	Low	High	(%)	*p*^1^
Age					0.21				0.96				0.06
25–69	121	39	82	(68)		49	72	(60)		38	83	(69)	
70–84	147	34	113	(77)		62	85	(58)		28	119	(81)	
85+	34	11	23	(68)		14	20	(59)		10	24	(71)	
FIGO					0.26				0.49				0.08
Ia	11	2	9	(82)		4	7	(64)		2	9	(82)	
Ib	37	16	21	(57)		19	18	(49)		16	21	(57)	
II	110	28	82	(75)		43	67	(61)		22	88	(80)	
III	121	32	89	(74)		46	75	(62)		30	91	(75)	
IV	19	5	14	(74)		10	9	(47)		5	14	(74)	
Not available	4												
Lymph node metastasis					0.42				0.97				0.54
None	137	41	96	(70)		55	82	(60)		36	101	(74)	
Unilateral	76	18	58	(76)		31	45	(59)		17	59	(78)	
Bilateral	34	12	22	(65)		13	21	(62)		11	23	(68)	
Not available	55												
Tumour diameter (cm)					0.001				0.002				0.001
0.3–2.5	92	34	58	(63)		50	42	(46)		33	59	(64)	
2.6–4.0	94	30	64	(68)		35	59	(63)		25	69	(73)	
4.1–20.0	100	14	86	(86)		30	70	(70)		12	88	(88)	
Not available	16												
Tumor differentiation					0.09				0.10				0.08
Well	76	27	49	(65)		39	37	(49)		25	51	(67)	
Moderate	154	43	111	(72)		61	93	(60)		39	115	(75)	
Poor	72	14	58	(81)		25	47	(65)		12	60	(83)	
Depth of invasion (mm)					0.01				0.001				0.007
0.0–4.0	80	32	48	(60)		45	35	(44)		30	50	(63)	
4.1–8.0	99	24	75	(76)		38	61	(62)		21	78	(79)	
8.1–40.0	112	24	88	(79)		34	78	(70)		21	91	(81)	
Not available	11												
Infiltration of vessel					0.33				0.50				0.41
No	233	67	166	(71)		99	134	(58)		61	172	(74)	
Yes	66	15	51	(77)		25	41	(62)		14	52	(79)	
Not available	3												
p14^2^					1.00				1.00				0.88
Low (-)	135	98	37	(27)		46	89	(66)		84	51	(38)	
High (+)	77	56	21	(27)		26	51	(66)		49	28	(36)	
Not available	100												
p16^2^					0.63				0.88				0.15
Low (< 5%)	148	40	108	(73)		51	97	(66)		37	111	(75)	
High (≥ 5%)	63	15	48	(76)		21	42	(67)		10	53	(84)	
Not available	91												
p21^2^					0.17				0.87				0.10
Low (-)	122	36	86	(71)		42	80	(66)		32	90	(74)	
High (+)	90	19	71	(79)		30	60	(67)		15	75	(84)	
Not available	90												
p27^2^					0.18				0.56				0.19
Low (< 50%)	164	39	125	(76)		54	110	(67)		33	131	(80)	
High (≥ 50%)	48	16	32	(67)		18	30	(63)		14	34	(71)	
Not available	90												
p53^2^					0.70				0.12				0.93
Low (< 5%)	93	25	68	(73)		26	67	(72)		20	73	(79)	
High (≥ 5%)	118	29	89	(75)		45	73	(62)		26	92	(78)	
Not available	91												
Cyclin A^2^					0.68				0.09				0.59
Low (< 5%)	61	17	44	(72)		26	35	(57)		15	46	(75)	
High (≥ 5%)	151	38	113	(75)		46	105	(70)		32	119	(79)	
Not available	90												
Cyclin D1^2^					0.87				0.75				0.83
Low (-)	156	40	116	(74)		52	104	(67)		34	122	(78)	
High (+)	56	15	41	(73)		20	36	(64)		13	43	(77)	
Not available	90												
Cyclin D3^2^					0.25				0.76				0.69
Low (< 50%)	153	43	110	(72)		51	102	(67)		35	118	(77)	
High (≥ 50%)	59	12	47	(80)		21	38	(64)		12	47	(80)	
Not available	90												
Cyclin E^2^					0.72				0.64				0.32
Low (< 50%)	173	44	129	(75)		60	113	(65)		36	137	(79)	
High (≥ 50%)	39	11	28	(72)		12	27	(69)		11	28	(72)	
Not available	90												
HPV^2^					0.87				0.47				0.98
Low (-)	168	44	124	(74)		57	111	(66)		39	129	(77)	
High (+)	44	11	33	(75)		15	29	(66)		8	36	(82)	
Not available	90												

^1 ^Pearson chi-square ^2 ^In previous reports, p53, p16, p21, p27, p14, cyclin A, cyclin D1, cyclin D3, cyclin E and HPV have been studied [28-31].

### Cell Line

Human vulvar squamous cell carcinoma cell lines SW-954 (ATCC, Manassas, VA, U.S.A) and CAL-39 (DSMZ, Germany) were grown as monolayer cultures recommended by the suppliers. For RNA analysis, cells from monolayer culture were harvested by using 0.01 M EDTA solution and washed in PBS. For immunohistochemstry, cells were fixed in 4% formalin and embedded in paraffin.

### Immunohistochemical method

Sections (4 μm) from formalin-fixed, paraffin-embedded tissue were processed for immunohistochemistry using the Dako EnVision™ + System, Peroxidase (DAB) (K4007, Dako Corporation, CA, U.S.A). Deparaffinized sections were microwaved in 10 mM citrate buffer pH 6.0 to unmask the epitopes and treated with 0.3% hydrogen peroxidase (H_2_O_2_) for 5 min to block endogenous peroxidase. Monoclonal 14-3-3σ antibody (clone 1433S01, 1:75, 2.7 μg IgG_1_/ml) from NeoMarkers, CA, U.S.A were applied on the sections for 30 min at room temperature. The primary antibody is highly specific to 14-3-3σ and shows no cross-reaction with other isoforms of 14-3-3 (information from NeoMarkers). Subsequently, the slides were incubated with horseradish peroxidase (HRP) labeled polymer conjugated goat-anti-mouse for 30 min, followed by incubation with 3'3-diaminobenzidine tetrahydrochloride (DAB) for 10 min. The sections were counterstained with hematoxylin, dehydrated and mounted in Diatex.

Normal skin was included as positive control in all series. Negative controls included substitution of the monoclonal antibody with mouse myeloma protein of the same subclass and concentration as the monoclonal antibody. Both nuclear and cytoplasmic staining was considered positive. Immunostaining was scored on a 3 – tiered scale for both intensity (absent/weak, 1; moderate, 2; strong, 3) and extent of staining (percentage of positive tumor cells: < 10%, 1; 10–50%, 2; > 50%, 3). Scoring results of intensity and extent were multiplied to give a composite score ranging from 1 to 9 for each section. Protein expression in cytoplasm was defined as high when composite scores were ≥ 6, and low when composite scores were < 6, whereas, protein expression in nucleus were classified as high when any 14-3-3σ staining was observed in the tumor and low when no staining was seen. This is based on staining pattern observed in normal vulvar epithelium. We later verified all other cutoff values. Examination of immunostaining was performed by two independent observers (Z.W. and R.H.) with no knowledge of patient outcome. All discordant scores were reviewed until final agreement was obtained. Reproducibility in the semiquantitative scoring was controlled. The kappa values were 0.72 for both extra- and intra-observer variability when determined on a subset of 40 patients.

### Laser captured microdissection (LMC)

Eleven vulvar carcinomas frozened at -70°C were selected for LMC and RNA isolation.

First, 8 μm frozen sections were fixed in ethanol, stained with hematoxylin-eosin (HE), dehydrated in graded ethanol and zylene and air-dried for 5 min. Then, by using a PixCell laser capture microscope (Arctrurus Engineering Inc., Mountain View, CA), 30–200 pure tumor cells were captured on caps by focal melting of the membrane through laser activation. The LMC parameters were as follows, a laser power of 70–80 mW, laser pulse duration of 1.2–3.5 ms, and laser spot size of 7.5–15 μm in diameter. Finally, the caps with the selected cells were covered with lysis buffer in Eppendorf tubes and rotated several times for homogenization before RNA isolation.

### RNA isolation

An Absolutely RNA™ Nanoprep Kit (Stratagene Inc. La Jolla, USA), which was special designed to purify RNA from 1 to 10^4 ^cells, was employed to extract and purify RNA from cells captured on caps by LCM. Total RNA and Protein Isolation Kit (Macherey-Nagel, Duren, Germany) was used to isolate total RNA form cell line SW-954 and CAL-39 as recommended by the manufacturer.

### RT-PCR

Transcriptor First Stand cDNA Synthesis Kit (Roche Inc., Mannheim, Germany) was used to synthesis cDNA. The total reaction volume was 20 μl and the reaction systems were: (1) RNA from LMC tissues: 4 μl 5 × Transcriptor RT Reaction Buffer, 0.5 μl Protector Rnase Inhibitor, 0.5 μl Transcriptor Reverse Transcriptase, 2 μl dNTP Mix (PCR grade), 1 μl Anchored Oligo (dt) _18 _Primer, 2 μl Water (PCR Grade), and 10 μl RNA. (2) RNA form cell line: 4 μl 5 × Transcriptor RT Reaction Buffer, 0.5 μl Protector Rnase Inhibitor, 0.5 μl Transcriptor Reverse Transcriptase, 2 μl dNTP Mix (PCR grade), 1 μl Anchored Oligo (dt)_18 _Primer, 10 μl Water (PCR Grade), and 2 μl RNA. The reaction condition was: 65°C for 10 min for mixture of cDNA and primer pre-denaturation, followed by 55°C for 30 min for reverse transcription of RNA, ended by 85°C for 5 min for inaction of reverse transcriptase.

### Quantitative Real-Time PCR

Five μl cDNA from LMC samples and 2 μl cDNA from cell lines were used as templates in subsequent Real-Time reaction performed on LightCycler 2.0 system (Roche Inc., Mannheim, Germany) using LightCycler^® ^TaqMan^® ^Master kit (Roche Inc., Mannheim, Germany) and gene specific Hydrolysis (TaqMan) Probes, according to the manufacturer's instruction. Glyceraldehyde-3-phosphate dehydrogenase (GAPDH) was used as an internal control for relative gene expression quantification and for each gene amplification. Cp values (reading obtained at 530 nm) were normalized to the Cp of the GAPDH using the LightCycler analysis software. The following sets of primers were used to amplify gene products for Real-Time PCR reactions: GAPDH forward-5'-AGC CAC ATC GCT CAG ACA, reverse-5'-GCC CAA TAC GAC CAA ATC C; 14-3-3σ forward-5'-GAC ACA GAG TCC GGC ATT G, reverse-5'-ATG GCT CTG GGG ACA CAC.

### Statistical analyses

The association between 14-3-3σ protein expression and clinicopathologic variables, and that between 14-3-3σ protein expression and 14-3-3σ mRNA expression levels were evaluated by the Person χ^2 ^test. The Kaplan and Meier method and the log-rank test were used to estimate and compare survival rate. Disease-specific survival was defined as the interval between diagnosis and death due to vulvar cancer. A Cox proportional hazards regression model was used for multivariate evaluation of survival rates. A backward stepwise regression with a *p *= 0.05 in univariate analysis as the inclusion criterion was used. All calculation was performed using the SPSS 15.0 statistical software package (SPSS, Chicago, IL). Statistical significance was considered as *p *≤ 0.05.

## Results

### 14-3-3σ protein expression

**Figure 1:**
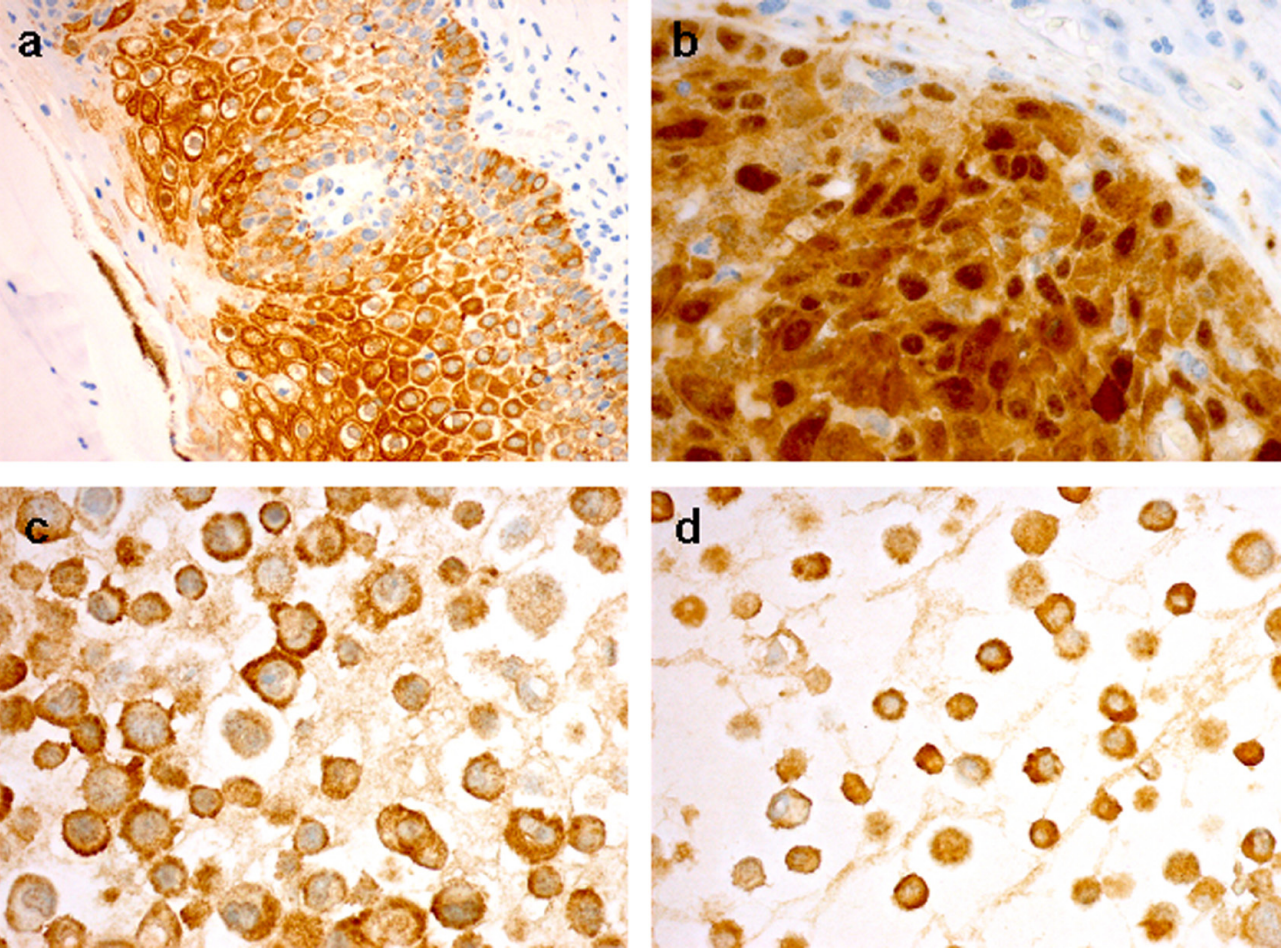
**Expression of 14-3-3σ protein**. Expression of 14-3-3σ protein in normal vulvar epithelium (a), vulvar carcinoma (b), SW-954 cell line (c) and CAL-39 cell line (d).

In normal vulvar epithelium, none of the 11 cases showed nuclear 14-3-3σ immunostaining. Nevertheless, high level of cytoplasmic staining (score ≥ 6) was present in 11/11 (100%) cases. The 14-3-3σ immunostaining was found in the basal, parabasal, middle and top layers of the vulvar epithelium (Figure 1a). In vulvar carcinomas, high 14-3-3σ expression (score ≥ 6) in cytoplasm was found in 218 (72%) cases and low levels in 84 (28%), whereas, high levels of nuclear immunostaining of 14-3-3σ (any positive nuclei) were observed in 177 (59%) cases, and low levels in 125 (41%). High 14-3-3σ expression (score ≥ 6) in cytoplasm/nuclei was found in 226 (75%) cases and low levels in 76 (25%) (Figure 1b and Table 2).

**Table 2:** Immunostaining results for 14-3-3σ

Score	Cytoplasm	Nucleus	Cytoplasm and nucleus
			
	n (%)	n (%)	n (%)
0	2 (0.7)	125 (41.4)	2 (0.7)
1	1 (0.3)	0 (0.0)	1 (0.3)
2	1 (0.3)	16 (5.3)	1 (0.3)
3	67 (22.2)	76 (25.2)	58 (19.2)
4	13 (4.3)	28 (9.3)	14 (4.6)
6	142 (47.0)	50 (16.6)	119 (39.4)
9	76 (25.2)	7 (2.3)	107 (35.4)

Total	302 (100.0)	302 (100.0)	302 (100.0)

In the vulvar carcinoma cell lines SW-954 and CAL-39 high levels (score ≥ 6) of cytoplasmic 14-3-3σ immunostaining were observed (Figure 1c and 1d).

### 14-3-3σ mRNA expression and association with 14-3-3σ protein levels

**Figure 2:**
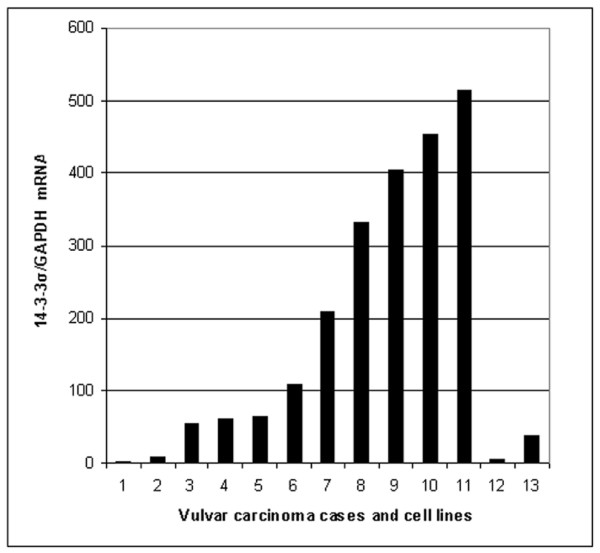
**Expression of 14-3-3σ mRNA in vulvar carcinomas and vulvar cell lines**. Expression of 14-3-3σ mRNA in vulvar carcinomas (no.1–11), vulvar carcinoma cell lines SW-954 (no.12) and CAL-39 (no.13).

Expression of 14-3-3σ mRNA was found in all laser-microdissection-captured vulvar carcinomas and in both vulvar carcinoma cell lines SW-954 and CAL-39. However, the mRNA expression levels were variable (Figure 2). No significant association was observed between levels of 14-3-3σ mRNA and 14-3-3σ protein expression in cytoplasm (*p *= 0.242), nucleus (*p *= 1.000) or cytoplasm/nucleus (*p *= 0.242) in vulvar carcinomas. Both cell lines showed low levels of 14-3-3σ mRNA expression and did not correlate to the high (score ≥ 6) 14-3-3σ protein expression.

### 14-3-3σ immunostaining in relation to clinicopathological parameters and patients survival

The levels of 14-3-3σ immunostaining in relation to clinicopathological parameters are shown in Table 1. High expression of 14-3-3σ in cytoplasm, nucleus and cytoplasm/nucleus were significantly correlated to large tumor diameter (*p *= 0.001, *p *= 0.002 and *p *= 0.001, respectively) and deep invasion (*p *= 0.01, *p *= 0.001 and *p *= 0.007, respectively). 14-3-3σ was neither associated to the cell cycle proteins p14, p16, p21, p27, p53 and cyclin A1, D1, D3 and E, nor to HPV.

In univariate analysis, with the variables defined in Table 1, age (*p *= 0.002), tumor diameter (*p *< 0.001), depth of invasion (*p *= 0.007), lymph node metastasis (*p *< 0.001), FIGO (*p *< 0.001) and infiltration of vessel (*p *< 0.001) were correlated to disease-specific survival. There was no significant association between disease-specific survival and 14-3-3σ expression in cytoplasm (*p *= 0.23), nuclear (*p *= 0.51) and cytoplasm/nuclear (*p *= 0.21). In multivariate analysis, FIGO, lymph node metastasis, age and depth of invasion were of independent prognostic significance (Table 3).

**Table 3:** Relative risk (RR) of dying from vulvar cancer

Variable	Grouping	Ratio of risk (RR)	95% CI	*p*
FIGO	1a vs 1b vs II vs III vs IV	1.86	1.18–2.9	0.007
Lymph nodes metastsis	Absent vs Present	1.49	1.00–2.23	0.05
Age	25–69 yrs vs 70–84 yrs vs = 85 yrs	1.10	1.00–1.04	0.05
Depth of invasion	0.0–4.0 mm vs 4.1–8.0 mm vs 8.1–35.0 mm	1.82	1.13–2.94	0.01

## Discussion

In our study normal vulvar squamous epithelium showed high cytoplasmic positivity for 14-3-3σ protein in the whole layer, which is similar to the findings in normal squamous epithelium of skin, esophagus and cervix [32]. However, in normal tongue 14-3-3σ immunoreactivity was often weak in the basal layer compared to that of the middle and top layers [32]. One explanation for these different results may be that p63 is extensively present in low layer of epidermal tongue [33], and that ΔNp63 depress 14-3-3σ expression [34] by controlling the p53-binding site of 14-3-3σ. However, when keratinocytes become more differentiated, and less ΔNp63 is produced, 14-3-3σ expression resume.

Mhawech et al [35] have studied the expression of 14-3-3σ protein in neoplastic tissues of urological and gynecological cancers. Positive 14-3-3σ protein expression was seen in urothelial bladder carcinoma (97.8%), squamous cell carcinoma of cervix (66.7%), endometrial adenocarcinoma (56.5%), prostatic adenocarcinoma (55.0%), renal papillary tumor (50%), ovarian adenocarcinoma (33.3%), and breast carcinoma (23.3%). In our study, positive 14-3-3σ staining was identified in cytoplasm and/or nucleus in 99.3% of vulvar carcinomas.

Gasco et al [25] identified methylation within the 14-3-3σ gene in approximately 60% of vulvar carcinomas. However, in the present study low levels of 14-3-3σ protein was found in only 25% of vulvar carcinomas. We have identified 14-3-3σ methylation in 57/57 (100%) vulvar carcinomas and 5/5 (100%) normal blood samples, whereas the two vulvar squamous cell carcinoma cell lines SW-954 and CAL-39 were unmethylated for 14-3-3σ (unpublished data). 14-3-3σ methylation in all normal blood samples and vulvar carcinomas may be due to the normal lymphocytes in blood and the lymphocytes surrounding the epithelial tumors. Previously, Bhatia et al [36] have demonstrated 14-3-3σ gene to be methylated in normal lymphocytes. Therefore, methylation identified within 14-3-3σ gene in some of the vulvar carcinomas reported by Gasco et al [25] may be due to inflammatory cells surrounding cancer cells [37]. Despite this problem, the low level of 14-3-3σ protein expression in a relative high number of vulvar carcinomas indicates that loss of 14-3-3σ expression may be important in the pathogenesis of a subset of vulvar squamous cell carcinoma.

Interestingly, we found different subcellular distribution of 14-3-3σ protein in cells in normal tissues and in cancer tissues. The high 14-3-3σ expression in cytoplasm but not in nucleus was found in all the 11 normal tissues, whereas, in vulvar carcinomas 72% and 59% of the cases had high expression of 14-3-3σ in cytoplasm and nucleus, respectively. Previously, Van Hermet and coworkers [38] showed that 14-3-3σ was mainly localized in the cytoplasm and only in lower levels present in the nucleus, which is consistent with subcellar distribution of the protein in normal vulvar cells. The cytoplasmic localization of 14-3-3σ protein in normal vulvar cells may prevent cyclin B1-CDC2 complex from entering the nucleus and initiate mitosis [14,39]. In the present work we have found in vulvar carcinoma cells that more 14-3-3σ protein moved from cytoplasm to nucleus and stayed there. This might imply that 14-3-3σ have less power to sequester cyclin B1-CDC2 complex in cytoplasm by its nucleus translocation, as a result cell fails to be arrested at G2 checkpoint, but enters into cell division. [14] Nuclear location of 14-3-3σ protein may thus play a role in tumorigenesis of vulvar squamous cell carcinomas. However, we found that high expression of 14-3-3σ in cytoplasm as well as in nucleus was significantly correlated to large tumor diameter and deep of invasion. Furthermore, neither 14-3-3σ expression in cytoplasm nor in nucleus was significant associated with clinical outcome. These results may indicate that the cellular localization of 14-3-3σ is not important for the progression of vulvar carcinomas. However, we cannot exclude the possibility that nuclear localization of 14-3-3σ protein may be an early event in carcinogenesis of vulvar carcinomas. Further studies are needed to clarify the importance of 14-3-3σ nuclear localization in development of vulvar carcinomas.

In our study, we found that high 14-3-3σ protein expression in cytoplasm, nuclear, and both cytoplasm/nuclear was significant correlated with large tumor diameter and deep invasion. This is in disagreement with the findings in colorectal carcinomas [24] and gastric carcinomas [40], where 14-3-3σ protein expression did not correlate with tumor size.

For the first time, 14-3-3σ protein expression and its prognostic importance has been examined in vulvar squamous cell carcinomas. No significant association was found between disease-specific survival and 14-3-3σ expression, which was in line with the findings in breast carcinomas [22]. However, in breast carcinomas 14-3-3σ cytoplasmic subcellular localization showed a statistically significant correlation with survival in univariate analysis, but not in multivariate analysis [22]. Previously, loss of the 14-3-3σ protein expression was correlated with poor prognosis in epithelial ovarian cancer in univariate analysis, but not in multivariate analysis [41]. Patients with endometrial adenocarcinoma absence of 14-3-3σ protein expression had a shorter overall survival in multivariate analysis [23]. In contrast to these findings, Perathoner et al [24] found that in colorectal carcinoma patients increased expression of 14-3-3σ protein were associated with poor survival in multivariate analysis. Taken together, these studies indicate that the role of 14-3-3σ is cancer specific.

In the present work, 14-3-3σ was not associated to p53 protein expression, which was similar to the findings in a variety of cancers from bladder, prostate, endometrium, ovarium, breast [37] and neuroendocrine lung tumors [21]. These findings indicate that the expression of 14-3-3σ may be regulated by other factors than p53.

To our knowledge, the relationship between levels of 14-3-3σ mRNA and protein expression in vulvar carcinomas has not been studied previously. Gasco et al [25] found a marked reduction or a complete absence of 14-3-3σ mRNA in 20 vulvar carcinomas with CpG methylation. However, in that study no data of corresponding 14-3-3σ protein levels were included [25]. In our work, no significant association was identified between levels of 14-3-3σ mRNA and protein expression. This was consistent with the results in uterine cervical cancer [42] and colon cancer cell lines [24] where expression of 14-3-3σ mRNA did not fully correspond to 14-3-3σ protein expression. These findings indicate that 14-3-3σ expression is also regulated at the post-transcriptional level. There may be modifications of mRNA stability and/or translational efficiency [43], or alternatively, some regulation at the post-translational level may exist [23,43]. In breast cancer cells post-translational regulation by estrogen-induced zinc finger protein, resulting in 14-3-3σ degradation has been reported [37].

## Conclusion

Low levels of 14-3-3σ protein expression in 25% of the cases may indicate that 14-3-3σ protein expression may be associated with tumorigenesis in a subset of vulvar squamous cell carcinomas. However 14-3-3σ protein expression was not associated with prognosis.

## Competing interests

The authors declare that they have no competing interests.

## Authors' contributions

ZW participated in the design of the study, carried out the microdissection, protein, mRNA, statistical and data analysis and draft the manuscript. CGT collected clinical data, participated in interpretation of data and helped to draft the manuscript. ZS participated in microdissection and mRNA analysis and interpretation and revised the manuscript critically. GT participated in microdissection and mRNA analysis and interpretation and revised the manuscript critically. GY was involved in the project design and manuscript revising. JMN performed systematic pathologic review of vulvar carcinomas and revised the manuscript critically. RH participated in the design of the study, protein, statistical and data analysis and helped to draft the manuscript. All authors read and approved the final manuscript.

## Pre-publication history

The pre-publication history for this paper can be accessed here:



## References

[B1] Coulter J, Gleeson N (2003). Local and regional recurrence of vulval cancer: management dilemmas. Best Pract Res Clin Obstet Gynaecol.

[B2] Jones RW, Baranyai J, Stables S (1997). Trends in squamous cell carcinoma of the vulva: the influence of vulvar intraepithelial neoplasia. Obstet Gynecol.

[B3] Messing MJ, Gallup DG (1995). Carcinoma of the vulva in young women. Obstet Gynecol.

[B4] Tyring SK (2003). Vulvar squamous cell carcinoma: guidelines for early diagnosis and treatment. Am J Obstet Gynecol.

[B5] Qi W, Liu X, Qiao D, Martinez JD (2005). Isoform-specific expression of 14-3-3 proteins in human lung cancer tissues. Int J Cancer.

[B6] Yaffe MB, Rittinger K, Volinia S, Caron PR, Aitken A, Leffers H (1997). The structural basis for 14-3-3:phosphopeptide binding specificity. cell.

[B7] Dougherty MK, Morrison DK (2004). Unlocking the code of 14-3-3. J Cell Sci.

[B8] Fu H, Subramanian RR, Masters SC (2000). 14-3-3 proteins: structure, function, and regulation. Annu Rev Pharmacol Toxicol.

[B9] Mackintosh C (2004). Dynamic interactions between 14-3-3 proteins and phosphoproteins regulate diverse cellular processes. Biochem J.

[B10] Hermeking H, Lengauer C, Polyak K, He TC, Zhang L, Thiagalingam S (1997). 14-3-3 sigma is a p53-regulated inhibitor of G2/M progression. Mol cell.

[B11] Lakin ND, Jackson SP (1999). Regulation of p53 in response to DNA damage. Oncogene.

[B12] Taylor WR, Stark GR (2001). Regulation of the G2/M transition by p53. Oncogene.

[B13] Laronga C, Yang HY, Neal C, Lee MH (2000). Association of the cyclin-dependent kinases and 14-3-3 sigma negatively regulates cell cycle progression. J Biol Chem.

[B14] Chan TA, Hermeking H, Lengauer C, Kinzler KW, Vogelstein B (1999). 14-3-3Sigma is required to prevent mitotic catastrophe after DNA damage. Nature.

[B15] Ferguson AT, Evron E, Umbricht CB, Pandita TK, Chan TA, Hermeking H (2000). High frequency of hypermethylation at the 14-3-3 sigma locus leads to gene silencing in breast cancer. Proc Natl Acad Sci USA.

[B16] Iwata N, Yamamoto H, Sasaki S, Itoh F, Suzuki H, Kikuchi T (2000). Frequent hypermethylation of CpG islands and loss of expression of the 14-3-3 sigma gene in human hepatocellular carcinoma. Oncogene.

[B17] Lodygin D, Yazdi AS, Sander CA, Herzinger T, Hermeking H (2003). Analysis of 14-3-3sigma expression in hyperproliferative skin diseases reveals selective loss associated with CpG-methylation in basal cell carcinoma. Oncogene.

[B18] Lodygin D, Diebold J, Hermeking H (2004). Prostate cancer is characterized by epigenetic silencing of 14-3-3sigma expression. Oncogene.

[B19] Moreira JM, Gromov P, Celis JE (2004). Expression of the tumor suppressor protein 14-3-3 sigma is down-regulated in invasive transitional cell carcinomas of the urinary bladder undergoing epithelial-to-mesenchymal transition. Mol Cell Proteomics.

[B20] Suzuki H, Itoh F, Toyota M, Kikuchi T, Kakiuchi H, Imai K (2000). Inactivation of the 14-3-3 sigma gene is associated with 5' CpG island hypermethylation in human cancers. Cancer Res.

[B21] Yatabe Y, Osada H, Tatematsu Y, Mitsudomi T, Takahashi T (2002). Decreased expression of 14-3-3sigma in neuroendocrine tumors is independent of origin and malignant potential. Oncogene.

[B22] Simpson PT, Gale T, Reis-Filho JS, Jones C, Parry S, Steele D (2004). Distribution and significance of 14-3-3sigma, a novel myoepithelial marker, in normal, benign, and malignant breast tissue. J Pathol.

[B23] Ito K, Suzuki T, Akahira J, Sakuma M, Saitou S, Okamoto S (2005). 14-3-3sigma in endometrial cancer – a possible prognostic marker in early-stage cancer. Clin Cancer Res.

[B24] Perathoner A, Pirkebner D, Brandacher G, Spizzo G, Stadlmann S, Obrist P (2005). 14-3-3sigma expression is an independent prognostic parameter for poor survival in colorectal carcinoma patients. Clin cancer Res.

[B25] Gasco M, Sullivan A, Repellin C, Brooks L, Farrell PJ, Tidy JA (2002). Coincident inactivation of 14-3-3sigma and p16INK4a is an early event in vulval squamous neoplasia. Oncogene.

[B26] Shepherd JH (1996). Cervical and vulva cancer: changes in FIGO definitions of staging. Br J Obstet Gynaecol.

[B27] WHO (2003). Pathology and Genetics Tumours of the Breast and Female Genital Organs. World Health Organization Classification of Tumors.

[B28] Knopp S, Bjorge T, Nesland JM, Trope C, Scheistroen M, Holm R (2004). p16INK4a and p21Waf1/Cip1 expression correlates with clinical outcome in vulvar carcinomas. Gynecol Oncol.

[B29] Knopp S, Bjorge T, Nesland JM, Trope C, Holm R (2005). Cyclins D1, D3, E, and A in vulvar carcinoma patients. Gynecol Oncol.

[B30] Knopp S, Nesland JM, Trope C, Holm R (2006). p14ARF, a prognostic predictor in HPV-negative vulvar carcinoma. Am J Clin Pathol.

[B31] Scheistroen M, Trope C, Kaern J, Pettersen EO, Alfsen GC, Nesland JM (1997). DNA ploidy and expression of p53 and C-erbB-2 in extramammary Paget's disease of the vulva. Gynecol Oncol.

[B32] Nakajima T, Shimooka H, Weixa P, Segawa A, Motegi A, Jian Z (2003). Immunohistochemical demonstration of 14-3-3 sigma protein in normal human tissues and lung cancers, and the preponderance of its strong expression in epithelial cells of squamous cell lineage. Pathol Int.

[B33] Kurokawa I, Mizutani H, Kusumoto K, Nishijima S, Tsujita-Kyutoku M, Shikata N (2006). Cytokeratin, filaggrin, and p63 expression in reepithelialization during human cutaneous wound healing. Wound Repair Regen.

[B34] Ghahary A, Marcoux Y, Karimi-Busheri F, Li Y, Tredget EE, Kilani RT (2005). Differentiated keratinocyte-releasable stratifin (14-3-3 sigma) stimulates MMP-1 expression in dermal fibroblasts. J Invest Dermatol.

[B35] Mhawech P, Greloz V, Assaly M, Herrmann F (2005). Immunohistochemical expression of 14-3-3 sigma protein in human urological and gynecological tumors using a multi-tumor microarray analysis. Pathol Int.

[B36] Bhatia K, Siraj AK, Hussain A, Bu R, Gutierrez MI (2003). The tumor suppressor gene 14-3-3 sigma is commonly methylated in normal and malignant lymphoid cells. Cancer Epidemiol Biomarkers Prev.

[B37] Mhawech P (2005). 14-3-3 proteins – an update. Cell Res.

[B38] van Hemert MJ, Niemantsverdriet M, Schmidt T, Backendorf C, Spaink HP (2004). Isoform-specific differences in rapid nucleocytoplasmic shuttling cause distinct subcellular distributions of 14-3-3 sigma and 14-3-3 zeta. J Cell Sci.

[B39] Piwnica-Worms H (1999). Cell cycle. Fools rush in. Nature.

[B40] Tanaka K, Hatada T, Kobayashi M, Mohri Y, Tonouchi H, Miki C (2004). The clinical implication of 14-3-3 sigma expression in primary gastrointestinal malignancy. Int J Oncol.

[B41] Akahira J, Sugihashi Y, Suzuki T, Ito K, Niikura H, Moriya T (2004). Decreased expression of 14-3-3 sigma is associated with advanced disease in human epithelial ovarian cancer: its correlation with aberrant DNA methylation. Clin cancer Res.

[B42] Sano T, Shimooka H, Weixa P, Segawa A, Jian Z, Motegi A (2004). Immunohistochemical expression of 14-3-3 sigma protein in various histological subtypes of uterine cervical cancers. Pathol Int.

[B43] Audic Y, Hartley RS (2004). Post-transcriptional regulation in cancer. Biol Cell.

